# Multiple surgical resections for progressive IDH wildtype glioblastoma—is it beneficial?

**DOI:** 10.1007/s00701-024-06025-x

**Published:** 2024-03-15

**Authors:** Susan Isabel Honeyman, William J. Owen, Juan Mier, Katya Marks, Sohani N. Dassanyake, Matthew J. Wood, Rory Fairhead, Pablo Martinez-Soler, Hussain Jasem, Ananya Yarlagadda, Joy R. Roach, Alexandros Boukas, Richard Stacey, Vasileios Apostolopoulos, Puneet Plaha

**Affiliations:** 1 Department of Neurosurgery, Oxford University Hospitals NHS Foundation Trust, Oxford, UK; 2 Nuffield Department of Surgery, University of Oxford, Oxford, UK; 3 Nuffield Department of Clinical Neurosciences, University of Oxford, Oxford, UK

**Keywords:** Recurrent, Glioblastoma, Repeat, Surgical resection

## Abstract

**Purpose:**

The role of repeat resection for recurrent glioblastoma (rGB) remains equivocal. This study aims to assess the overall survival and complications rates of single or repeat resection for rGB.

**Methods:**

A single-centre retrospective review of all patients with IDH-wildtype glioblastoma managed surgically, between January 2014 and January 2022, was carried out. Patient survival and factors influencing prognosis were analysed, using Kaplan–Meier and Cox regression methods.

**Results:**

Four hundred thirty-two patients were included, of whom 329 underwent single resection, 83 had two resections and 20 patients underwent three resections. Median OS (mOS) in the cohort who underwent a single operation was 13.7 months (95% CI: 12.7–14.7 months). The mOS was observed to be extended in patients who underwent second or third-time resection, at 22.9 months and 44.7 months respectively (*p* < 0.001). On second operation achieving > 95% resection or residual tumour volume of < 2.25 cc was significantly associated with prolonged survival. There was no significant difference in overall complication rates between primary versus second (*p* = 0.973) or third-time resections (*p* = 0.312). The use of diffusion tensor imaging (DTI) guided resection was associated with reduced post-operative neurological deficit (RR 0.37, *p* = 0.002), as was use of intraoperative ultrasound (iUSS) (RR 0.45, *p* = 0.04).

**Conclusions:**

This study demonstrates potential prolongation of survival for rGB patients undergoing repeat resection, without significant increase in complication rates with repeat resections. Achieving a more complete repeat resection improved survival. Moreover, the use of intraoperative imaging adjuncts can maximise tumour resection, whilst minimising the risk of neurological deficit.

## Introduction

Glioblastoma is the most common and aggressive primary neoplasm of the brain [6]. Disease prognosis remains poor, with a median life expectancy of between 14 and 18 months [25]. The optimal management for newly diagnosed glioblastoma is maximal surgical resection, with concomitant radiotherapy and Temozolomide, followed by six cycles of adjuvant Temozolomide chemotherapy [31]. Studies have also demonstrated that maximising surgical resection improves patient survival [18, 19]. Despite advances in treatment, the infiltrative nature of the disease inevitably results in tumour recurrence and progression.

There is currently no standardised management for recurrent glioblastoma (rGB) following primary resection, but treatment options include repeat irradiation, chemotherapy, immunotherapy or reoperation. Repeat resection aims to reduce tumour volume to delay symptoms progression, maintain quality of life and prolong survival [3, 4]. However, the current evidence for the impact of repeat surgery on morbidity and survival remains equivocal [14, 38]. In this study, we aim to assess (1) the impact of second or third-time resections on patient survival in recurrent IDH wildtype glioblastoma; (2) other factors impacting overall survival; (3) the impact of extent of second or third resection achieved on survival; (4) the impact of the timing of repeat resections on survival; (5) the risks of re-operation through assessment of post-operative complications; and (6) the impact of intra-operative adjuncts on the extent of resection and operative safety.

## Methods

### Study design

Patients who underwent surgical resection of glioblastomas at the John Radcliffe, Oxford University Hospital, between January 2014 and January 2022, were identified through a retrospective search of the hospital’s surgical database. Oxford University Hospitals NHS Foundation Trust Research and Development department and Health Research Authority approval was received for data analysis (IRAS No: 256310).

Patients were included only if they were (1) diagnosed with WHO grade 4 glioblastoma on initial tumour tissue diagnosis, (2) had isocitrate dehydrogenase (IDH) wildtype disease and (3) underwent primary surgical resection of the tumour after diagnosis. Patients were excluded if they had (1) secondary, IDH mutant glioblastoma; (2) underwent non-surgical management of disease at diagnosis (chemotherapy or radiotherapy); (3) underwent a biopsy as the primary operative management; (4) underwent emergency glioblastoma resection; (5) initially underwent non-surgical management of tumour recurrence (chemotherapy or radiotherapy); and (6) were lost to follow-up.

Patient records were retrospectively reviewed. Data was extracted on patient gender, age, presenting symptoms, date of diagnosis (based on initial CT or MRI scan), tumour location, WHO performance status at diagnosis, co-morbidities, histological diagnosis, O^6^-methylguanine-DNA methyl-transferase (MGMT) promoter methylation status, date of each surgical resection, intraoperative use of 5-aminolevulinic acid (5ALA), intraoperative ultrasound (iUSS), pre-operative diffusion tensor imaging (DTI) and post-operative complications (assessed at hospital discharge and clinic follow-up). The extent of resection achieved was recorded using tumour volumes calculated from Brainlab Software based on the presence of any residual contrast enhancing tumour on post-operative T1-weighted MRI. Complete or gross total resection (GTR) was defined as 100% resection of contrast enhancing tumour. Near total resection (NTR) was defined as > 95% resection of contrast enhancing tumour, whilst subtotal resection (STR) was defined as < 95% resection of contrast enhancing tumour. Neurological deficits were stratified into those persisting > 30 days following the operation and transient deficits, which had resolved by 30 days follow-up. Details were collected on adjuvant chemotherapy, radiotherapy regimens and finally date of patient death.

### Statistical analysis

Overall survival (OS) was defined as the time between the radiological diagnosis, on the first CT or MRI scan, and date of death. Patients with unknown survival status were censored at the last date of follow-up. All statistical analyses were performed using software from SPSS Statistics 21, IBM, Chicago IL, USA. Kaplan–Meier method analysis was used to assess the survival differences between population subgroups, and the significance of differences in survival was analysed using pairwise Log-rank testing. Univariate and multivariate analyses were carried out using Cox proportional hazards models to assess the impact of other potential prognostic factors on survival. Analyses were either parametric or nonparametric, depending on data attributes and assessment of normality using Shapiro–Wilk test. Data are presented as mean ± 95% confidence interval, or median with interquartile range as appropriate. Parametric data were compared using paired or unpaired Student’s *t* test. Non-parametric data were compared using the Wilcoxon rank sum (unpaired) or signed rank (paired) tests. Statistical significance was set at *p* < 0.05.

## Results

### Patient cohort

Four hundred eighty-five patients were identified who underwent surgical resection of an IDH wild-type glioblastoma between January 2014 and January 2022. Fifty-three patients were excluded due to incomplete data or loss to follow-up. As a result, 432 patients were included in the final analysis. Then, 329 patients had a single resection, 83 underwent second operation for recurrent disease and 20 received a further third-time resection. The median age at diagnosis was 61 years (range 23–82 years). Of the patients, 281 were male (65.0%) and 151 female (35.0%). After primary resection, most individuals received adjuvant chemoradiotherapy (356/394, 90.4%). Patient demographics, surgical details and chemoradiotherapy regimens are summarised in Table 1.

**Table 1:** Summary of patient baseline demographics and treatment characteristics

Variable	All patients (*n* = 432)	1 Resection (*n* = 329)	2 Resections (*n* = 83)	3 Resections (*n* = 20)
Age				
< 70 years	351	251	80	20
> 70 years	81	78	3	0
Gender				
Male	281	220	51	10
Female	151	109	32	10
WHO performance status				
PS = 0–1	401	310	73	18
PS = 2–3	31	19	10	2
MGMT Status				
Unmethylated	171	122	43	6
Methylated < 10%	96	76	15	5
Methylated 10–25%	25	18	6	1
Methylated > 25%	40	28	8	4
Not performed	100	85	11	4
Operative details for initial resection				
5-ALA used	374	281	74	19
DTI used	335	259	64	12
iUSS used	161	131	26	4
Extent of resection				
GTR (100%)	110	68	41	4
NTR (> 95%)	160	95	48	7
STR (< 95)	272	234	25	13
Chemo-radiotherapy				
RT 60/30 + TMZ	278	186	74	18
RT 40/15 + TMZ	24	19	4	1
RT 60/30 alone	21	18	3	0
RT 40/15 alone	29	28	1	0
RT 30/15 alone	4	4	0	0
No CRT	38	36	1	1
Unknown	38	38	0	0

### Pathology

All patients were IDH-1 mutation negative. MGMT promotor methylation status was analysed in 332 patients (76.8%): 171 patients had unmethylated disease, < 10% methylation was seen in 96 patients, 10–25% methylation in 25 patients and > 25% methylation in 40 patients.

### Intraoperative imaging adjuncts

Neuro-navigation was used for all patients. In most patients, intraoperative adjuncts were also used to guide resection, with 374/432 receiving 5-ALA, for 335/432 patients pre-operative DTI was obtained and in 161/432 patients iUSS was used.

### Patient survival

#### Patient survival

**Fig. 1:**
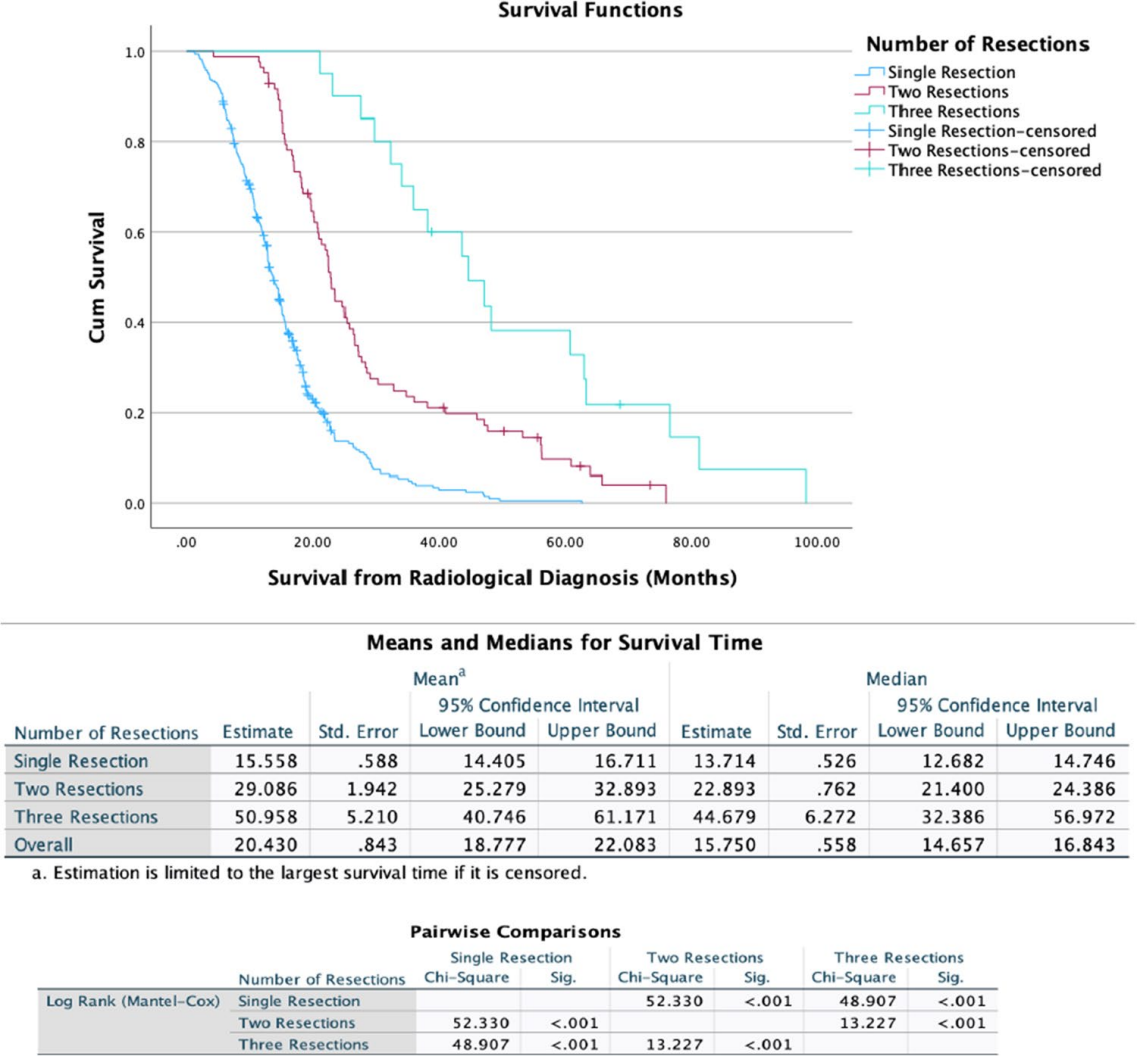
Kaplan–Meier Curves demonstrating overall survival in patients who underwent either single, two- or three-time resections for glioblastoma

Figure 1 shows the Kaplan–Meier survival curves for patients stratified by number of resections. Median OS (mOS) in the cohort who underwent a single operation was 13.7 months (95% CI 12.7–14.7 months). The mOS was prolonged in patients who underwent second or third-time resection, at 22.9 months (95% CI: 21.4–24.4 months) and 44.7 months (95% CI: 32.4–57.0 months), respectively (*p* < 0.001).

#### Other factors associated with overall survival

**Fig. 2:**
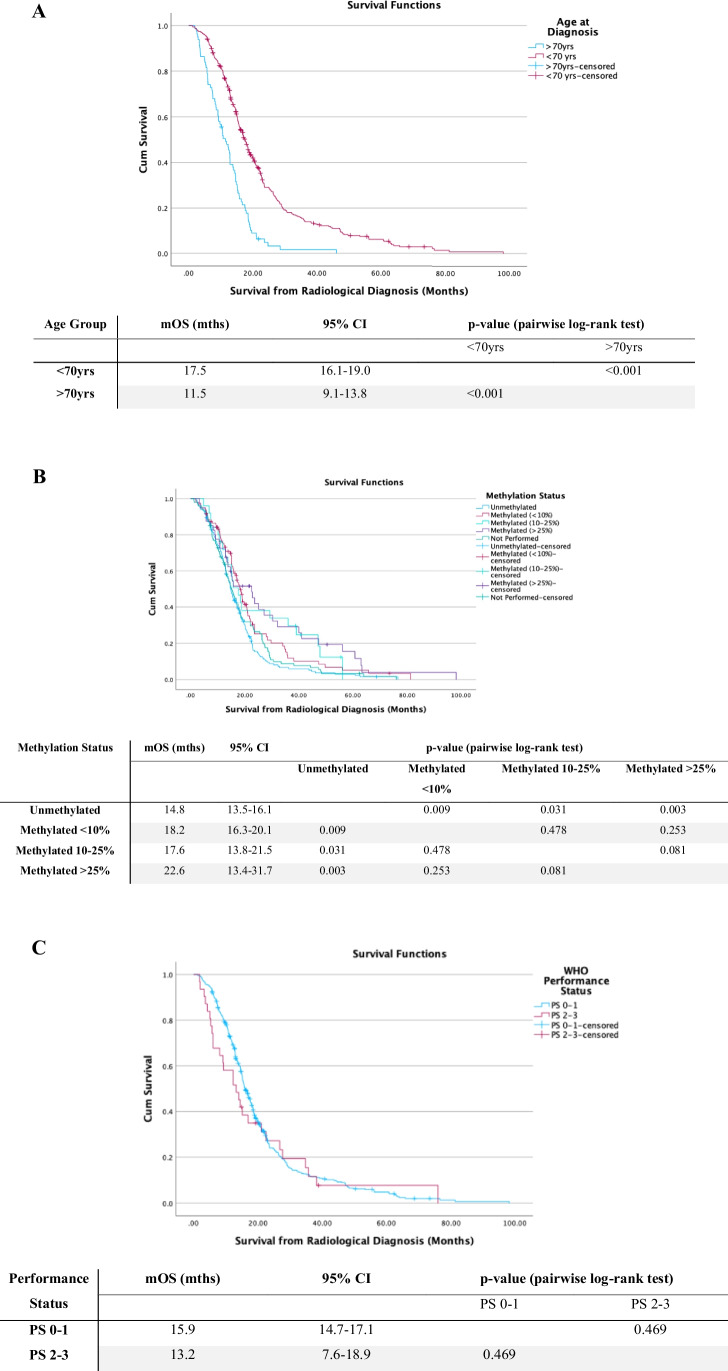
Kaplan–Meier curves for survival from radiological diagnosis. **A** Stratified for age > 70 years or < 70 years. **B** Stratified by MGMT status and **C** stratified for baseline WHO performance status. Survival distributions were compared using pairwise log-rank test. *mOS* median overall survival, *95%CI* 95% confidence intervals

Other factors were also associated with increased survival. Significant improvement in mOS was observed in patients < 70 years of age at time of diagnosis, compared with those > 70 years (Fig. 2A, p < 0.001). Similarly, patients with methylated MGMT promoter had improved mOS (Fig. 2B). A lower WHO performance status at time of diagnosis was associated with improved survival but was not statistically significant (Fig. 2C, p = 0.469). The use of intraoperative imaging adjuncts including 5-ALA, DTI and iUSS also did not significantly impact survival (*p* > 0.05).

### Extent of resection and overall survival

**Fig. 3:**
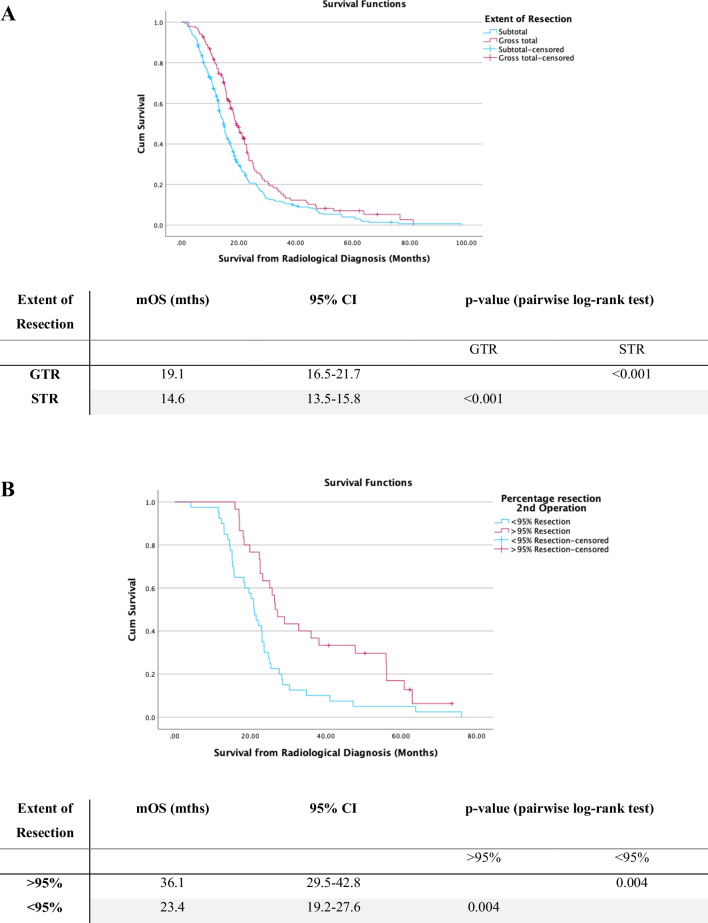

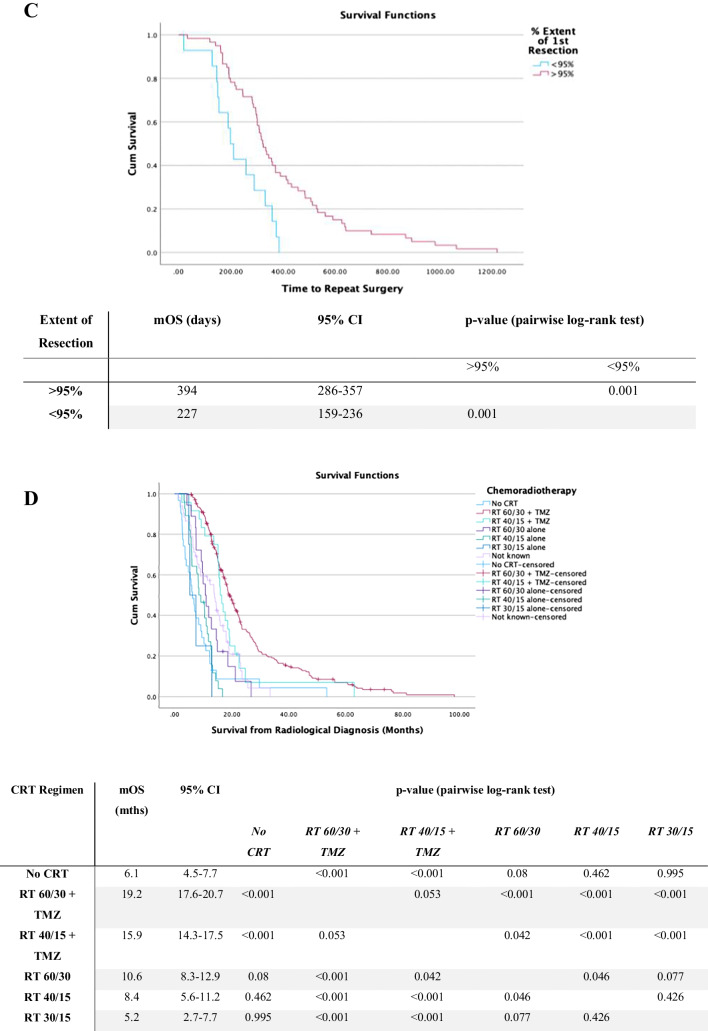
Kaplan–Meier curves for survival from radiological diagnosis. **A** Stratified by extent of resection of primary operation. **B** Stratified by extent of resection on second operation. **C** Kaplan–Meier demonstrating time to repeat operation in days stratified by extent of resection on primary operation. **D** survival from radiological diagnosis stratified for chemoradiotherapy regimen. Survival distributions were compared using pairwise log-rank test. mOS, median overall survival; 95%CI, 95% confidence intervals

Maximising resection on first operation significantly improved survival with mOS with > 95% resection of 19.1 months (95% CI:16.5–21.7 months) in comparison to 14.6 months with STR (95% CI:13.5–15.8 months, *p* < 0.001) (see Fig. 3A). Cox regression analysis found that on second operation achieving > 95% resection in comparison to < 95% resection was a significant predictor for prolonged survival. The mOS with > 95% resection on second operation was 36.1 months (95% CI: 29.5–42.8 months) versus mOS of 23.4 months (95% CI: 19.2–27.6 months) with < 95% resection (*p* = 0.004) (see Fig. 3B). It was also found that a residual volume of < 2.25 cc versus > 2.25 cc was a predictor for prolonged survival on second resection. The mOS with > 2.25 cc residual was 20.6 months (95% CI: 13.7–27.9) and for < 2.25 cc residual was 34.1 months (95% CI: 28.7–39.5) (*p* < 0.001). For third time operation, no extent of resection or residual volume was a statistically significant predictor of survival. At third operation < 95% resection mOS 50.4 months (95% CI: 28.5–60.8 months) and > 95% resection mOS 62.7 months (95% CI: 17.3–66.7 months) (*p* = 0.644).

#### Timing of repeat surgery

The median time between first and second resection was 11.0 months. Eighty-seven patients underwent repeat resection > 6 months following primary operation, whilst 16 had repeat resection at < 6 months. There was no significant difference in survival between cohorts who had repeat resection < 6 months vs. > 6 months following first surgery (*p* = 0.170). However, patients who underwent < 95% resection on initial operation in comparison to > 95% resection had significantly earlier repeat resections (7.4 months vs. 13.0 months following primary resection, *p* = 0.001) see (Fig. 3C).

#### Oncological treatment and overall survival

With respect to oncological managements, there was no significant difference in mOS between patients receiving adjuvant Temozolomide (TMZ) in combination with standard (RT60/30 + concomitant TMZ) versus hypo-fractionate dose (RT40/15 + TMZ) radiotherapy (*p* = 0.053). In comparison to radiotherapy alone, both RT60/30 + TMZ and RT40/15 + TMZ demonstrated significant increase in survival (*p* < 0.05). Patients who received no chemoradiotherapy (CRT) had a significantly reduced survival of 6.1 months (95% CI: 4.5–7.7 months, *p* < 0.001). Survival with respect to CRT regimen is shown in Fig. 3D.

#### Multivariate analysis for overall survival

The direct survival impact of each variable was evaluated using Cox proportional hazards model (Table 2).

**Table 2:** Univariate and multivariate Cox-proportional hazards regression analysis of co-variates on survival

Variable	Univariate analysis		Multivariate analysis	
	HR (95% CI)	*P* value	HR (95% CI)	*P* value
Age				
< 70 years	0.53 (0.41–0.69)	< 0.001	0.67 (0.49–0.91)	0.010
> 70 years				
Gender				
Male	1.21 (0.98–1.50)	0.73	1.01 (0.81–1.26)	0.960
Female				
WHO performance status	1.48 (0.99–2.20)	0.054	1.32 (0.88–1.98)	0.187
MGMT status				
Methylated < 10%	0.69 (0.52–0.91)	0.009	0.76 (0.57–1.01)	0.054
Methylated 10*–*25%	0.58 (0.37–0.93)	0.024	0.61 (0.38–0.97)	0.036
Methylated > 25%	0.54 (0.37–0.80)	0.002	0.58 (0.39–0.86)	0.007
Unmethylated				
Extent of resection				
> 95% resection	0.69 (0.55–0.87)	< 0.001	0.71 (0.56–0.90)	0.004
< 95% resection				
DTI	0.99 (0.78–1.25)	0.912	0.89 (0.66–1.21)	0.465
5-ALA	0.93 (0.76–1.14)	0.502	1.09 (0.84–1.43)	0.508
iUSS	1.09 (0.89–1.35)	0.406	1.04 (0.82–1.32)	0.758
Chemo*–*radiotherapy				
RT 60/30 + TMZ	0.22 (0.15–0.32)	< 0.001	0.19 (0.13–0.29)	< 0.001
RT 40/15 + TMZ	0.33 (0.19–0.57)	< 0.001	0.23 (0.13–0.42)	< 0.001
RT 60/30 alone	0.65 (0.36–1.19)	0.161	0.32 (0.17–0.59)	< 0.001
RT 40/15 alone	1.31 (0.77–2.22)	0.325	0.63 (0.36–1.12)	0.115
RT 30/15 alone	1.92 (0.67–5.48)	0.226	1.13 (0.38–3.35)	0.828
No CRT				
Repeat resection				
1 Resection				
2 Resections	0.37 (0.28–0.49)	< 0.001	0.41 (0.30–0.55)	< 0.001
3 Resections	0.15 (0.09–0.25)	< 0.001	0.16 (0.09–0.28)	< 0.001

Following adjustment for gender, age, WHO performance status, MGMT status, extent of resection, use of intraoperative imaging adjuncts (5-ALA, DTI and iUSS) and CRT regimen, repeat surgical resection continued to demonstrate significant increase in survival (*p* < 0.001) (see Table 2).

### Predictors at primary resection of eligibility for repeat resection

The median pre-operative tumour volume at time of first operation for patients who underwent repeat operation was 26.2 cm^3^, in comparison to 22.4 cm^3^ for patients who underwent a single operation (Wilcoxon, *p* = 0.655). The median percentage primary resection for patients who had a single operation was 95.9%, in comparison to 98.2% for patients who underwent repeat operation (Wilcoxon, *p* = 0.624). The median residual volume was 0.31 cm^3^ for patients who underwent a single resection versus 0.20 cm^3^ for patients who underwent a repeat resection (Wilcoxon, *p* = 0.818). Proportionally there were similar numbers of eloquent tumours (those located in or adjacent to (1) Broca’s area, (2) Wernicke’s area, (3) primary sensory cortex and (4) primary motor cortex) between the two cohorts. Further, 91/329 (27.7%) patients who underwent single resection and 32/103 (31.1%) patients who underwent repeat operation had eloquent tumours. There was no significant difference in pre-operative tumour volumes, percentage extent of resection, residual tumour volume or eloquence of tumour location that might predict the patients who would go on to have repeat resection at time of primary operation.

**Fig. 4:**
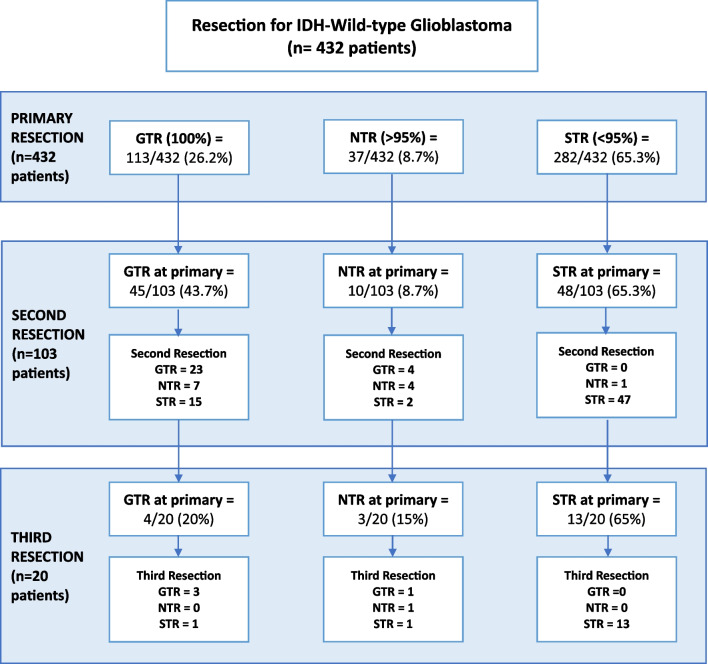
Flowchart summarising the completeness of resection achieved at primary operation and then subsequent resections. (Gross total resection (GTR) = 100%. Near total resection (NTR) =  > 95%. Subtotal resection (STR) =  < 95%.)

GTR was achieved in 68/329 (20.7%) patients who underwent a single operation. For patients who underwent more than one resection, at initial operation GTR was achieved in 42/103 (40.7%) of patients. This is illustrated in Fig. 4. The odds of going on to have a repeat tumour resection were significantly greater with GTR at initial resection, compared with < 100% resection (OR 2.64, 95%CI: 1.64–4.24, *p* = 0.0001). Then, > 95% resection was achieved in 95/329 (28.9%) who had a single operation and in 55/103 (53.3%) who went on to have repeat operations. There was a significantly increased chance of going on to have a repeat tumour resection with > 95% resection versus STR at initial operation (OR 2.82, 95%CI: 1.79–4.44, *p* < 0.0001). Our data suggests that a more complete primary resection increased the chance of patients going on to have repeat resections.

### Complications of repeat surgery

There was no significant difference in overall complication rates between primary (12.5%) and secondary resections (12.6%) (RR 0.99, 95% CI 0.56–1.74, *p* = 0.973). Similarly, there was no significant increased risk of complications with third time resection, in comparison to single resection (RR 1.60, 95% CI: 0.64 to 3.98, *p* = 0.312). At > 30 days post-operatively, 6.5% of patients had neurological deficit following primary resection, in comparison to 5.8% on secondary resection (RR 0.899, 95% CI: 0.38–2.11, *p* = 0.806). There was however statistically significant increased risk of neurological deficit on third time versus primary resection (RR 3.08, 95% CI: 1.20–7.95, *p* = 0.020). There was no significant difference in infection rates between primary and secondary resection (RR 1.14, 95% CI: 0.476–2.749, *p* = 0.764). Complications are summarised in Table 3. Notably, the use of pre-operative DTI to guide resection significantly reduced the risk of post-operative neurological deficit (RR 0.37, 95% CI: 0.20–0.69, *p* = 0.002), as did use of iUSS (RR 0.45, 95% CI: 0.21–0.96, *p* = 0.04) (see Table 4). There was no surgery related mortality.

**Table 3:** Table summarising the complications of each resection

Complications	1ST Resection (*N* = 32)	2ND Resection (*N* = 103)	3RD Resection (*N* = 20)
Infection	22/432	6/103	0/20
CSF leak	7/432	2/103	0/20
Neurological			
Weakness	11/432	2/103	2/20
(Temporary speech deficit)	32/432	3/103	0/20
Lasting Speech deficit	11/432	3/103	2/20
Visual deficit	6/432	1/103	0/20
Haematoma requiring evacuation	2/432	0/103	0/20
Overall patient complications	54/432 (12.5%)	13/103 (12.6%)	4/20 (20.0%)

**Table 4:** Table summarising impact of intraoperative adjuncts on post-operative neurological deficit in observed following resections

Adjunct	Post-operative neurological deficit	No post-operative neurological defict	Relative risk (95% CI)	*P* value
DTI				
DTI USED (***N*** = 446)	18/446	432/446	0.36 (0.18–0.73)	0.005
DTI NOT USED (***N*** = 109)	12/109	97/109		
IUSS				
IUSS USED (***N*** = 210)	5/210	205/210	0.37 (0.14–0.95)	0.039
IUSS NOT USED (***N*** = 325)	23/355	332/355		

## Discussion

This retrospective series demonstrates that repeat resection for IDH wild-type glioblastoma prolongs patient survival, whilst overall complication rates are not dissimilar to those at primary resection.

Achieving a more complete resection at primary operation increases the likelihood of going on to have repeat operation and, as is already established, is associated with prolonged survival. Undergoing repeat operation for rGB was associated with survival benefit. The use of intraoperative adjuncts including DTI and USS was associated with reduced risk of neurological deficits.

### The impact of repeat resection on overall survival

The benefit of repeat resection for rGB remains equivocal, with no large-scale prospective studies comparing survival following repeat surgeries and guidance on patient selection is yet to be established. In our study of 432 patients, 329 had 1 resection, 83 underwent 2 resections and 20 received 3 resections. Reported rate of re-operation for rGB ranges from 8% [20] to 29% [22]. In our patient series, 23.8% of patients received repeat surgery. At last follow-up, the mOS for our patients who underwent 1, 2 or 3 resections was 13.7, 22.9 and 44.7 months, respectively. Each repeat resection statistically significantly prolonged patient survival (*p* < 0.001). Following multivariate analysis, controlling for other prognostic factors, this effect persisted. The results of our review support suggestions that repeat resection for rGB can improve patient survival.

Other patient series suggest survival benefit is conferred by reoperation [7, 15, 22, 28]. A retrospective, multi-centre study of 503 patients who underwent repeat resection for rGB demonstrated median survival was prolonged to 22.7 months and 29.3 months, with second and third resections respectively [28]. Similarly, Chaichana et al. [7] found on multivariate analysis when controlling for age, neurological function, extent of resection and adjuvant therapies, patients who underwent 1, 2 or 3 resections had a median survival of 4.5, 16.2 and 24.4 months, respectively (*p* < 0.05). Montemurro et al. [21] carried out a literature review evaluating evidence for reoperation in rGB. In their analysis of 28 studies including 2279 patients, they demonstrated that mOS from diagnosis, for patients who underwent repeat resection, was 18.5 months. Each of these studies support the view that repeat resection prolongs patient survival. Patient age, performance status and completeness of resection achieved were also important predictors of survival [21, 28]. Other series have not replicated these reports of prolonged survival with repeat resection [8, 14]. Evidence on the survival benefit of repeat resection in summarised in Table 5.

**Table 5:** Table summarising literature evaluating the survival benefit conferred by repeat resection for recurrent glioblastoma

Study	Study design	Total number of patients	Number of resections	Number of patients	Median survival (months)	Notes
Azoulay et al. [2]	Retrospective	136	1	68	5.3	
			2	68	9.6	
Chaichana et al. [22]	Retrospective	578	1	354	6.8	Risk of infections or iatrogenic deficits did not increase with repeat resections (*p* > 0.05)
			2	121	15.5	
			3	41	22.4	
			4	15	26.6	
Delgado-Fernandez et al. [9]	Retrospective	121	1	90	/	The reoperation group had a median increase survival of 6.4 months compared with the non-reoperation group (*p* < 0.001)
			2	31	/	
Djamel-Eddine et al. [11]	Retrospective	132	1	68	11	
			2	53	16	
			3	11	18	
Filippini et al. [13]	Retrospective	676	1	503	/	Multivariable analysis showed no effect of reoperation on survival, whether performed within 9 months of the first surgery (HR 0.86, *p* 5 0.256) or after 9 months (HR 0.98; *p* 5 0.860)
			2	173	/	
Goldman et al. [14]	Retrospective	163	1	74	/	When timing of repeat resection was ignored, repeat resection was associated with a lower risk of death (HR 0.62, *p* = 0.01). However, when timing was taken into account, repeat resection was associated with a higher risk of death (HR 2.19, *p* < 0.001)
			2	89	/	
Mukherjee et al. [28]	Prospective	312	1	167	6.9	Complication rates were 5.5% and 6.2% following repeat resection and primary resection, respectively (*p* > 0.05)
			2	145	10.8	
Nava et al. [23]	Prospective	764	1	368	/	HR for death after recurrence—1.05 (0.71–1.54) with reoperation vs conservative management
			2	138	/	
Ortega et al. [24]	Retrospective	202	1	83	21.1	After adjusting for age, multiple resections were not an independent predictor of survival
			2	94	25.5	
			3	25	29.0	
Ringel et al. [15]	Retrospective	503	1	82	22.7	
			2	421	29.3	
			3	72	34.3	
			4	9	26.4	
Sastry et al. [30]	Retrospective	368	1	291	7.0	
			2	77	12.8	
Suchorska et al. [32]	Prospective	105	1	34	/	Post-recurrence survival (PRS) was 11.4 months (95% CI: 8.4–12.3) in patients who underwent surgery versus 9.8 months (95% CI: 6.6–15.1) in patients who did not undergo surgery (P 0.633)
			2	71	/	
Tugcu et al. [33]	Retrospective	50	1	39	6.9	
			2	11	9.6	
Tully et al. [34]	Retrospective	204	1	105	9.0	
			2	49	20.1	
Woernle et al. [37]	Retrospective	98	1	58	14.8	
			2	40	18.8	
Wann et al. [36]	Retrospective	120	1	60	14	
			2	60	22	

### Predictors at primary resection of eligibility for repeat resection

We analysed baseline factors from primary resection that might be predictors of patients who would be more eligible to go on to have repeat operations. There was no significant difference in pre-operative tumour volumes, percentage extent of resection, residual tumour volume or eloquence of tumour location between the patient cohorts who underwent a single versus multiple resections for rGB. However, our data did suggest that the more complete the primary resection, the more likely patients are to be candidates to go on to have a repeat resection. This further builds upon well-established understanding that greater extent of primary resection for glioblastoma is associated with an improved overall survival and progression free survival for patients [18, 19]. Our results further emphasise the importance of optimising completeness of resection at primary operation.

### What is a meaningful second tumour resection?

An unanswered question for surgeons when considering second resection is extent of tumour resection required to provide better tumour control and improve prognosis. The impact of extent of resection at repeat operation is not well established. From our analyses, achieving > 95% resection or a residual tumour volume or < 2.25 cc was a significant predictor of prolonged survival. For third time operation no percentage of resection or residual volume was found to be a statistically significant predictor of survival, but it must be acknowledged our sample size was small (*n* = 20). Bloch et al. [5] have also assess the respective impact of GTR (defined as > 95% resection) versus STR (< 95% resection) on survival. They found GTR at repeat operation to be an independent positive predictor of survival. Meanwhile, extent of initial resection was not a statistically significant factor when repeat extent of resection was included in the model, suggesting that GTR at second craniotomy could overcome the effect of an initial STR.

### Timing of second surgery?

Another key question in the management of glioblastoma is timing of repeat surgery. There is no evidence base at present on when and at which volume of recurrence to consider repeat surgery for rGBM. From our series, we found no difference in mOS in the cohort who had repeat surgery whilst on adjuvant TMZ treatment < 6 month from primary resection vs patients who had surgery > 6 months following primary resection. Goldman et al. (2018) conducted a time-dependent analysis of repeat resection. When timing was ignored, repeat resection was associated with prolonged survival. However, when timing was considered, repeat resection was associated with a higher risk of death (HR:2.19, *p* < 0.001) [14]. It is not clear if, as discussed in this paper, patients with poorer risk factors were more likely to be offered and to receive repeat resection, such that the differences in survival may be due to underlying risk factors rather than to repeat resection itself. Moreover, it is notable that in this series the median time between primary and secondary resection was 7.7 months, potentially suggesting more patients underwent an early repeat operation. In our study, median time to repeat operation was 11.0 months, with few patients undergoing early reoperation (< 6 months, *n* = 16).

### Repeat resection versus complication rates

The potential survival benefits of repeat resection must be balanced against the risk of morbidity and complications. There is concern that with repeat resection risks of neurological deficit might be increased. Ringel et al. in their series of 503 patients who underwent repeat resection found increased rates of complications with subsequent surgeries [28]. At initial surgery, 5.1% of patients acquires new neurological deficit, whilst following repeat resection this increased to 7.6%. Hoover et al. [17] also demonstrated increased risk of complications with repeat cranial surgeries, with neurological complications occurring in 4.8% of patients at first surgery, 12.1% at second, 8.2% at third and 11.1% at 4 or more surgeries. Contrastingly, Mukherjee et al. found no significant difference in complications rates between primary and subsequent GB resections [22]. In our series, the > 30-day neurological deficit following primary resection for GB was 6.5%, in comparison to 5.8% following a second operation. There was no significant difference in overall complications rates between primary and secondary resections (*p* = 0.973), or indeed between first and third-time resections (*p* = 0.312).

### Complication rates and surgical adjuncts

In terms of mitigating the risk of complications with repeat resection, we noted the use of operative imaging adjuncts, in addition to neuro-navigation, might reduce complication rates. DTI-guided resection reduced the risk of new neurological deficit by 63% (*p* = 0.002). Through DTI tractography it is possible to map white matter fibre tracts in vivo, to aid in selection and planning of oncological treatment [35]. DTI can assist in pre-operative planning through identifying patients who are high risk candidates for repeat operation, based on the anatomic subcortical white matter tracts in spatial relation to the tumour. It can also aid in the planning of a surgical corridor to maximise extent tumour of resection, whilst avoiding damage to eloquent white matter tracts [12]. DTI can provide crucial information in rGB surgery as tracts can be displaced and distorted by tumour, making them difficult to locate based on anatomical knowledge alone.

The use of iUSS was also associated with reduced risk of surgical complications (RR 0.45, 95% CI: 0.21–0.96, *p* = 0.04). During tumour resection, pre-operative image guidance degrades in accuracy due to brain shift and anatomical deformation during surgery [10]. iUSS can assist in real-time tumour localisation and facilitate differentiation of tumour from surrounding normal parenchyma, reducing chances of functional deficit but also risk of leaving residual tumour [26]. We would advocate for the use of both DTI and iUSS in the repeat resection of rGB, to minimise the risk of neurological deficit. The benefit of neuronavigation, 5-ALA, DTI and iUSS for GB is being investigated in a NIHR funded multi-centre randomised trial (FUTURE-GB) [27].

### Strengths and limitations

The role of repeat resection in rGB remains poorly understood. Much of the evidence currently available on survival benefit of repeat resection for rGB has been obtained through literature-based comparison [1, 16] or includes heterogenous patient groups, including together varying IDH subtypes and anaplastic astrocytomas [1, 29]. This makes survival benefits in specific patient cohorts difficult to delineate.

This present study affords a large series of patients, all of whom had wild-type IDH glioblastoma disease, who underwent standardised perioperative care in a single neurosurgical centre. Risk of confounders was minimised as there were no significant differences between those who underwent single and repeat resections in terms of age, methylation status or adjuvant chemo-radiotherapy regimen received.

Certain limitations of this study must however be acknowledged. This was a retrospective review of cases, meaning there was no pre-defined criteria for the selection of patients who should undergo repeat resection. A significant challenge in the investigation of rGB resection is the inherent selection bias, with those who are selected for reoperation being those who are stronger surgical candidates with better physiological reserves. There was also no standardisation in terms of the use of intraoperative adjuncts such as iUSS and DTI. This study did not match or control for pre-operative predictors of surgical success, which could mean overestimation in the benefits of repeat resection. Finally, the number of patients who underwent three resections was small (*n* = 20), making it difficult to draw conclusions about the true benefit of further resection in this subgroup.

## Conclusions

This study demonstrates potential prolongation of survival for rGB patients who undergo repeat tumour resection. Specifically, achieving > 95% resection or a residual volume of < 2.25 cc on repeat operation confers statistically significant survival benefit. Timing of repeat resection was not shown to impact survival. Importantly, there was no significant increase in complication rates observed between initial and repeat resections. Moreover, the use of intraoperative imaging adjuncts, including DTI and iUSS, can maximise tumour resection whilst minimising risk of neurological function.

## Data Availability

The data presented in this study is available on reasonable request to the corresponding author. The data are not publicly available due to the data-sharing policies of our institution.
